# Effects of moxibustion on autophagy and ferroptosis in a disease-syndrome combination rat model of rheumatoid arthritis

**DOI:** 10.3389/fmed.2026.1908133

**Published:** 2026-08-17

**Authors:** Jiajia Sang, Zixiang Jin, Hui Zuo, Long Zeng, Yi Yue, Meng Wang, Jun Hu, Feng Hao

**Affiliations:** 1 Department of Tuina, Affiliated Hospital of Nanjing University of Chinese Medicine, Nanjing, China; 2 School of Acupuncture-Moxibustion and Tuina, School of Health Preservation and Rehabilitation, Nanjing University of Chinese Medicine, Nanjing, China; 3 Department of Integrated Traditional Chinese and Western Medicine, Peking University First Hospital, Beijing, China; 4 Institute of Basic Theory of Chinese Medicine, China Academy of Chinese Medical Sciences, Beijing, China

**Keywords:** autophagy, ferroptosis, moxibustion, rheumatoid arthritis (RA), synovitis

## Abstract

Rheumatoid arthritis (RA) is a chronic autoimmune disease characterized by persistent synovial inflammation, fibroblast-like synoviocyte hyperplasia, and progressive cartilage and bone destruction. Moxibustion is widely used as a non-pharmacological adjunctive intervention for RA; however, its specific therapeutic effects and its associations with autophagy and ferroptosis remain insufficiently characterized. This protocol describes a randomized, controlled, assessor-blinded preclinical study designed to evaluate moxibustion in a disease-syndrome combination rat model of RA. Eligible rats will undergo exposure to a controlled wind-, cold-, and dampness-related environment followed by Complete Freund's Adjuvant (CFA) injection. After successful model induction has been confirmed using prespecified criteria, animals will be randomly allocated to a normal control group, an untreated model group, a heat-matched sham-moxibustion group, a Zusanli (ST36) moxibustion group, and a methotrexate active-comparator group. All animals will undergo standardized handling and restraint procedures. Moxibustion will be administered for 20 min once daily for 15 consecutive days, whereas methotrexate will be administered at the prespecified dose and frequency. The primary outcome will be the histopathological synovitis score. Secondary outcomes will include paw volume, arthritis index, joint structural damage, serum and synovial inflammatory mediators, and functional assessments. Autophagy will be evaluated using LC3-II/LC3-I, SQSTM1/p62, Beclin-1, and complementary assessments of autophagic flux. Ferroptosis-related outcomes will include GPX4, SLC7A11, ACSL4, lipid peroxidation, glutathione, reactive oxygen species, and tissue iron levels. Outcome assessment and statistical analysis will be performed by investigators blinded to group allocation. This study is expected to provide a reproducible framework for distinguishing the specific effects of moxibustion from non-specific heat, restraint, and handling effects, and to generate mechanistic evidence supporting subsequent translational and clinical investigations.

## 1 Introduction

Rheumatoid arthritis (RA) is a chronic autoimmune disease primarily characterized by persistent synovial inflammation, abnormal hyperplasia of synovial tissue, and irreversible destruction of cartilage and bone (1, 2). In Traditional Chinese Medicine (TCM), RA is classified under the category of “Bi Syndrome”. It is attributed to a deficiency of vital energy (Qi) combined with the invasion of pathogenic factors such as wind, cold, dampness, and heat. Over time, the disease evolves into a complex condition involving a mixture of deficiency and excess, making it difficult to cure. Moxibustion, an external therapeutic modality of TCM, has been widely used throughout history for the treatment of RA.

Moxibustion involves the combustion of moxa wool near specific acupoints, generating thermal and non-thermal stimulation. Clinical trials have evaluated moxibustion as an adjunct to conventional pharmacological treatment for RA, with some studies reporting improvements in pain, disease activity, joint symptoms, and functional outcomes (3–5). However, systematic reviews have emphasized the heterogeneity, limited sample sizes, and risk of bias in the available evidence, precluding definitive conclusions regarding its clinical efficacy (3, 4). Methotrexate remains a central component of conventional disease-modifying antirheumatic therapy (6, 7). Nevertheless, incomplete treatment responses, adverse effects, and persistent pain or functional impairment have prompted interest in complementary non-pharmacological interventions.

Autophagy and ferroptosis are increasingly recognized as interconnected cellular processes involved in synovial homeostasis. Dysregulated autophagy may promote the survival and persistence of pathogenic fibroblast-like synoviocytes (8, 9). Ferroptosis is an iron-dependent form of regulated cell death driven by phospholipid peroxidation. Ferroptosis susceptibility varies among synovial fibroblast subpopulations and may influence synovial hyperplasia, inflammation, and tissue destruction in RA (10).

Previous studies conducted by our group and other investigators have suggested that moxibustion may influence inflammatory cytokines, autophagy-related signaling, synovial-cell survival, and ferroptosis-associated lipid metabolism in experimental arthritis (11–15). These earlier studies provide the rationale for the present protocol but are independent of the proposed experiment. The present study is designed prospectively and differs from previous work by incorporating prespecified outcomes, a heat-matched sham intervention, standardized restraint procedures, blinded outcome assessment, and an integrated evaluation of autophagy and ferroptosis. No data generated from the study described in this protocol have been reported in the present manuscript.

The primary objective of this study is to determine whether ST36 moxibustion reduces histopathological synovitis in a disease-syndrome combination rat model of RA compared with untreated and heat-matched sham controls. Secondary objectives are to compare moxibustion with methotrexate and to characterize moxibustion-associated alterations in inflammatory mediators, autophagic flux, and ferroptosis-related pathways. We hypothesize that ST36 moxibustion will reduce synovial inflammation and joint damage and that these effects will be accompanied by coordinated regulation of autophagy- and ferroptosis-related processes.

## 2 Methods and analysis

### 2.1 Animals, sample size, and housing conditions

A total of 50 healthy female Sprague–Dawley rats of specific-pathogen-free grade, aged 5 weeks and weighing 200–220 g, will be obtained from Shanghai Lingchang Biotechnology Co., Ltd., Shanghai, China. The animals will be housed in individually ventilated cages within a specific-pathogen-free barrier facility at 21 ± 2 °C, 50% ± 5% relative humidity, and a 12-h light/dark cycle. Five rats will be housed per cage and provided with cobalt-60-irradiated maintenance feed and sterile drinking water *ad libitum*. Cages will be changed twice weekly, and nesting paper or appropriate toys will be provided as environmental enrichment. After a minimum acclimatization period of 7 days, the rats will be allocated to five experimental groups, with 10 rats in each group. The individual rat will be regarded as the experimental unit. Multiple tissue sections, microscopic fields, or technical replicates obtained from the same animal will be averaged before statistical analysis and will not be treated as independent biological replicates.

The planned sample size of 10 rats per group is based on the animal allocation in the approved project protocol and the group sizes used in the research team's previous experiments. Nevertheless, an *a priori* sample-size calculation based on the primary outcome, namely the histopathological synovitis score, will be completed before the formal experiment. A two-sided α level of 0.05, a statistical power of 80%, and an anticipated attrition rate of 10% will be applied. If the calculated sample size differs from the planned number, the animal number will be adjusted after ethical approval. The experimental design and reporting plan were developed with reference to the ARRIVE 2.0 and PREPARE guidelines (16, 17).

### 2.2 Experimental design, grouping, randomization, and blinding

This study is designed as a randomized, controlled, parallel-group, assessor-blinded preclinical experiment. Animals will be allocated to the following five groups:

1. **Normal control group:** rats will receive neither environmental exposure nor Complete Freund's Adjuvant (CFA) injection;
2. **Untreated RA model group:** rats will undergo model induction but will receive no active therapeutic intervention;
3. **Heat-matched sham-moxibustion group:** modeled rats will receive temperature-matched electrical heat stimulation at Zusanli (ST36) without moxa combustion;
4. **Moxibustion group:** modeled rats will receive suspended moxibustion at ST36;
5. **Methotrexate group:** modeled rats will receive methotrexate as an active pharmacological comparator.

All animals will undergo standardized daily handling and restraint for 20 min. Therefore, a separate restraint-only group will not be established. The untreated RA model group will undergo the same restraint procedure as the intervention groups but will receive neither heat stimulation, moxibustion, nor methotrexate.

After successful model induction has been confirmed, eligible rats will be stratified according to body weight and baseline arthritis severity and randomly allocated to the experimental groups using a computer-generated randomization sequence. The allocation sequence will be generated and retained by an investigator who is not involved in intervention administration or outcome assessment.

Because of the nature of the intervention, the investigator administering moxibustion cannot be blinded. However, investigators responsible for paw-volume measurement, arthritis scoring, histopathological assessment, biochemical assays, image analysis, and statistical analysis will remain blinded to group allocation. Tissue samples and data files will be identified using anonymous numeric codes until the primary statistical analysis has been completed.

### 2.3 Establishment and evaluation of the disease-syndrome combination model

A disease-syndrome combination RA model will be established by combining exposure to wind-, cold-, and dampness-related environmental conditions with CFA-induced adjuvant arthritis, according to the research team's previously established procedure (18–21).

Following acclimatization, rats assigned to the modeling groups will be placed in a custom-made aluminum-alloy and transparent glass chamber for 20 consecutive days. Environmental exposure will be conducted for 12 h per day, from 20:00 to 08:00. An ultrasonic atomizer will be used to control humidity, and an appropriate quantity of ice will be placed in the chamber to maintain the temperature at 6 ± 2 °C. Relative humidity will be maintained at 80%−90%, and airflow will be generated using an electric fan operated at a standardized high setting.

Temperature and relative humidity will be monitored using calibrated digital sensors throughout each exposure session. The dimensions of the chamber, number of animals placed in the chamber during each session, fan model and position, and temperature- and humidity-recording intervals will be documented before the formal experiment.

On day 21, the plantar surface of the right hind paw will be disinfected with 75% ethanol. Under brief inhalational anesthesia induced with 4% isoflurane in oxygen and maintained at 2%−3% via a facemask, with adequate anesthetic depth confirmed by the absence of the pedal-withdrawal reflex 0.15 mL CFA obtained from Aladdin Biochemical Technology Co., Ltd., Shanghai, China (catalog No. F393378-10 mL) will be injected into the plantar region of the right hind paw (21). The normal-control rats will receive an equivalent volume of sterile saline at the corresponding site but will not undergo wind-, cold-, and dampness-related environmental exposure.

The rats will be observed for 3 days after CFA injection. Acute inflammatory swelling of the injected paw and ankle will be assessed 24 h after injection. The presence of redness, swelling, or inflammatory nodules involving the non-injected limbs, ears, or tail will also be recorded.

Paw volume will be measured using a calibrated plethysmometer 1 day before CFA injection, every 3 days after CFA injection, and at the end of the experiment. Paw-swelling percentage will be calculated as follows:

Paw swelling (%) = (Vt – Vn)/Vn × 100%

where *Vn* represents paw volume before CFA injection and *Vt* represents paw volume at the corresponding post-injection time point.

Arthritis severity will be evaluated every 3 days using the three non-injected limbs. Each limb will be graded using the following 0–4 scale:0, no erythema or swelling;

- 0, no erythema or swelling;
- 1, erythema or swelling involving the small toe joints;
- 2, swelling involving the toe joints and plantar region;
- 3, swelling involving the paw below the ankle;
- 4, swelling involving the entire paw, including the ankle.

The scores for the three non-injected limbs will be summed to obtain a total arthritis index ranging from 0 to 12 for each rat (21, 22). The intervention will begin 3 days after CFA injection and will therefore be described as an early therapeutic intervention in adjuvant arthritis, rather than treatment initiated after fully established chronic systemic polyarthritis.

### 2.4 Interventions

#### 2.4.1 Moxibustion intervention

During treatment, rats will be gently secured on a custom-made suspension wooden frame that allows stable positioning without excessive compression of the limbs, thorax, or abdomen.

Pure moxa sticks measuring 1 cm in diameter will be obtained from Wolong Hanyi Moxa Factory, Nanyang, China. ST36 will be located according to the standard rat acupoint atlas, approximately 5 mm distal to the head of the fibula on the posterolateral aspect of the knee. Hair surrounding the acupoint will be removed, and the acupoint will be marked before treatment.

Beginning 3 days after CFA injection, suspended moxibustion will be administered at unilateral ST36. The left and right ST36 points will be used on alternating days. The burning tip of the moxa stick will be positioned approximately 2 cm above the skin. Each session will last 20 min and will be performed once daily for 15 consecutive days (12, 18, 19).

The skin-surface temperature at ST36 will be monitored using an infrared thermal camera or calibrated thermocouple. Because the original project protocol standardized treatment primarily by distance rather than skin temperature, a pilot standardization procedure will be conducted to determine the target skin-temperature range before the formal experiment. Treatment duration, treatment distance, mean and maximum skin temperatures, moxa-stick consumption, operator identity, and adverse events will be recorded, because moxibustion temperature varies substantially with treatment distance, skin-surface temperature will be monitored prospectively rather than inferred solely from the nominal 2-cm distance (23, 24).

Moxa ash will be removed regularly to maintain a consistent treatment distance. The intervention will be conducted in a well-ventilated room. Rats will be monitored for excessive struggling, respiratory irritation, erythema, blistering, or burns.

#### 2.4.2 Heat-matched sham-moxibustion

Rats in the heat-matched sham-moxibustion group will be placed in the same restraint apparatus and treated at ST36 for 20 min once daily for 15 consecutive days.

A controllable electrical heating device will be positioned above ST36 without burning moxa. The device will be calibrated to reproduce the skin-surface temperature profile observed during active moxibustion. Treatment duration, acupoint location, alternating laterality, restraint procedure, operator contact, and room conditions will be identical to those of the active moxibustion group (24).

#### 2.4.3 Pharmacological comparator

Methotrexate will be used as an active pharmacological comparator. MTX will be purchased from Shanghai Pharmaceutical Co., Ltd., Shanghai, China; the catalog and batch numbers will be recorded before use. MTX will be suspended in 0.5% sodium carboxymethyl cellulose and administered by oral gavage at 0.5 mg/kg once every 3 days for 15 days, at an administration volume of 5 ml/kg. Animals in the other groups will receive an equivalent volume of 0.5% sodium carboxymethyl cellulose by oral gavage using the same schedule (25, 26).

### 2.5 Outcome measures

The primary outcome will be the histopathological synovitis score of the ankle joint at the end of the 15-day intervention. Secondary outcomes will include paw volume, arthritis index, synovial histopathological changes, synovial-cell ultrastructure, inflammatory cytokines, and molecular and biochemical indicators associated with autophagy and ferroptosis.

#### 2.5.1 Clinical and histopathological assessments

Paw volume and arthritis index will be assessed 1 day before CFA injection, every 3 days after CFA injection, and at the end of the experiment.

Approximately 24 h after the final intervention, three to six representative joint specimens will be collected from different pathological regions of each rat where technically feasible. Joint specimens will be fixed in 10% neutral-buffered formalin for 72 h and decalcified for 48 h using a nitric acid—formalin decalcifying solution. Samples will then undergo routine dehydration, paraffin embedding, and serial sectioning at a thickness of 5 μm. Sections will be stained with hematoxylin and eosin.

Histopathological assessment will include:

- Synovial lining-cell proliferation;
- Vascular proliferation;
- Fibrous-tissue proliferation;
- Lymphocyte and inflammatory-cell infiltration;
- Pannus formation;
- Cartilage and bone erosion.

A prespecified semiquantitative scoring system will be used. Two investigators blinded to group allocation will independently evaluate the sections, and discrepancies will be resolved by consensus or assessment by a third blinded investigator.

#### 2.5.2 Autophagy- and ferroptosis-related assessments

Transmission electron microscopy will be used to evaluate autophagosomes and mitochondrial ultrastructure in synovial fibroblast-like cells.

Autophagy-related changes will be assessed by measuring BECN1, LC3-II, ULK1, ATG3, ATG5, ATG12, and SQSTM1/p62, where applicable (12, 14, 18, 19, 27). Ferroptosis-related assessments will include Fe and Fe^2+^ concentrations, intracellular and lipid reactive oxygen species, glutathione and malondialdehyde levels, mitochondrial morphology, and the expression of GPX4, SLC7A11, ACSL4, LPCAT3, and ferritin (8, 9). Because static measurements of LC3 or BECN1 alone cannot distinguish increased autophagosome formation from impaired autophagosome degradation, the results will be described as changes in autophagy-related markers unless a lysosomal-inhibition or another validated flux assay is incorporated.

Ferroptosis-related assessments will include:

- Fe and Fe^2+^ concentrations measured using an Iron Assay Kit;
- Intracellular reactive oxygen species measured using flow cytometry;
- Lipid-peroxidation products measured using Liperfluo;
- Protein expression of BECN1, SLC7A11, GPX4, ferritin, and COX-2 measured using Western blotting;
- Glutathione-related indicators measured using an appropriate biochemical assay;
- Mitochondrial morphology assessed using transmission electron microscopy.

Ferroptosis will be evaluated using a combination of iron accumulation, lipid peroxidation, antioxidant-defense markers, and mitochondrial morphology rather than on the basis of a single protein marker (8, 9).

### 2.6 Animal welfare, sample collection, and humane endpoints

Animal welfare will be assessed at least twice daily during environmental exposure, after CFA injection, and throughout the intervention period. Monitoring will include body weight, food and water intake, posture, coat condition, spontaneous activity, respiratory status, gait, paw integrity, and signs of persistent pain or distress.

Prespecified humane endpoints will include:

- Body-weight loss of 20% or more relative to baseline;
- Persistent loss of appetite or food intake below 50% of the normal amount;
- Persistent inability to access food or water;
- Inability to stand or ambulate normally;
- Severe hypothermia or respiratory distress;
- Persistent recumbency;
- Paw necrosis, severe ulceration, infection, or self-mutilation;
- Major bleeding or severe dysfunction of the cardiovascular, respiratory, gastrointestinal, or nervous system;
- Severe pain or distress unresponsive to approved supportive treatment.

Animals reaching a humane endpoint will be euthanized immediately and will not be maintained until the scheduled study endpoint.

Approximately 24 h after the final intervention, rats will be deeply anesthetized using sodium pentobarbital (50 mg/kg, intraperitoneally), with adequate anesthetic depth confirmed by the absence of the pedal-withdrawal reflex. Blood will be collected through the abdominal aorta and centrifuged to obtain serum (25, 28). Animals will then be euthanized by carbon dioxide inhalation in accordance with the approved animal-welfare protocol, followed by confirmation of death. The joint synovial tissue will be carefully separated. One portion will be fixed for histopathological assessment, one portion will be prepared for transmission electron microscopy, and the remaining tissue will be snap-frozen in liquid nitrogen and stored at −80 °C for biochemical and molecular analyses.

### 2.7 Statistical analysis

Statistical analyses will be performed using SPSS version 23.0 (IBM Corp., Armonk, NY, USA). All tests will be two-sided, and *P* < 0.05 will be considered statistically significant. Effect estimates will be reported with 95% confidence intervals where appropriate.

Repeated measurements of paw volume, arthritis index, ankle diameter, and body weight will be analyzed using mixed-effects models or repeated-measures analysis of variance, with treatment group, time, and the group-by-time interaction included in the model.

Normally distributed terminal outcomes will be analyzed using one-way analysis of variance followed by Tukey's or Dunnett's multiple-comparison test. When the assumptions of normality or equal variance are not met, Welch's analysis of variance or the Kruskal–Wallis test followed by an appropriate *post hoc* test will be used.

The following comparisons will be specified prospectively:

- Moxibustion vs. untreated RA model;
- Moxibustion vs. heat-matched sham moxibustion;
- Methotrexate vs. untreated RA model;
- Moxibustion vs. methotrexate as an exploratory comparison.

No animal or observation will be excluded solely because it appears to be a statistical outlier. Any exclusion will be based on prespecified biological or technical criteria before group allocation is unblinded. Missing data and reasons for animal attrition will be reported for each group.

## 3 Discussion

This protocol describes a randomized and assessor-blinded preclinical study designed to evaluate the effects of ST36 moxibustion in a disease-syndrome combination rat model of RA. The study focuses on synovial inflammation and the potential involvement of autophagy- and ferroptosis-related processes.

A principal strength of the proposed design is the inclusion of a heat-matched sham-moxibustion group together with standardized handling and restraint procedures across all experimental groups. This design will help distinguish the effects associated with the complete moxibustion procedure from those caused by operator handling, restraint, or non-specific thermal stimulation. Methotrexate is included as an active pharmacological comparator rather than as a substitute for an appropriate procedural control. The combined assessment of clinical arthritis severity, synovial histopathology, inflammatory cytokines, and molecular indicators may provide complementary information regarding the biological responses associated with moxibustion. However, changes in autophagy- or ferroptosis-related markers will not, by themselves, establish that these pathways causally mediate the effects of moxibustion. Subsequent studies incorporating pathway-specific inhibitors, activators, or rescue experiments will be required to establish causal mechanisms.

Several limitations should be acknowledged. First, the wind-, cold-, and dampness-related environmental exposure and CFA injection model reproduces selected inflammatory and TCM syndrome-related features but cannot fully represent the genetic, immunological, and clinical heterogeneity of human RA. Second, investigators administering moxibustion cannot be blinded. Third, heat-only stimulation cannot completely reproduce all sensory, thermal, and chemical components of moxa combustion. Fourth, the inclusion of one animal sex may limit the generalizability of the findings if both male and female animals are not examined.

The findings of this preclinical study should not be interpreted as evidence that moxibustion can replace methotrexate or other disease-modifying antirheumatic drugs. Instead, the study is intended to provide mechanistic and methodological evidence supporting the evaluation of moxibustion as a potential adjunctive intervention. Further studies should evaluate treatment-temperature relationships, sex-dependent effects, interactions between moxibustion and conventional pharmacotherapy, and the reproducibility of the findings across laboratories.

## 4 Ethics and dissemination

The underlying disease-syndrome combination model and moxibustion procedures used in the preliminary animal experiment were reviewed and approved by the Experimental Animal Ethics Committee of Nanjing Damuda Pharmaceutical Co., Ltd. (approval no. IACUC-20231101). The experimental facility was licensed under SYXK (Su) 2022-0038.

Because the present prospective protocol incorporates a heat-matched sham-moxibustion group, a methotrexate active-comparator group, standardized restraint procedures, and a prespecified sample-size calculation, an ethical amendment or new animal ethics approval covering the final group allocation, animal number, anesthesia, analgesia, and euthanasia procedures will be obtained before the formal experiment begins.

All procedures will be conducted in accordance with applicable national regulations, institutional animal-welfare policies, and the ARRIVE 2.0 guidelines. The principles of Replacement, Reduction, and Refinement will be applied throughout the study. Any protocol amendment affecting animal numbers, model induction, intervention procedures, anesthesia, analgesia, or euthanasia will be submitted for ethical review before implementation.

The findings will be submitted to a peer-reviewed journal and presented at relevant scientific conferences, regardless of the direction or statistical significance of the results. De-identified raw data and the statistical analysis code will be made available after publication, subject to institutional and ethical requirements.
